# Adverse Hemodynamic Conditions Associated with Mechanical Heart Valve Leaflet Immobility

**DOI:** 10.3390/bioengineering5030074

**Published:** 2018-09-16

**Authors:** Fardin Khalili, Peshala P. T. Gamage, Richard H. Sandler, Hansen A. Mansy

**Affiliations:** 1 Biomedical Acoustics Research Laboratory, University of Central Florida, 4000 Central Florida Blvd, Orlando, FL 32816, USA; peshala@knights.ucf.edu (P.P.T.G.); rhsandler@gmail.com (R.H.S.); hansen.mansy@ucf.edu (H.A.M.); 2 Department of Mechanical Engineering, Embry-Riddle Aeronautical University, 600 South Clyde Morris Blvd., Daytona Beach, FL 32114-3900, USA; 3 College of Medicine, University of Central Florida, 6850 Lake Nona Blvd, Orlando, FL 32827, USA

**Keywords:** computational fluid dynamics, bileaflet mechanical heart valve, adverse hemodynamics, transvalvular pressure gradients, turbulent shear stresses, blood damage, platelet activation

## Abstract

Artificial heart valves may dysfunction, leading to thrombus and/or pannus formations. Computational fluid dynamics is a promising tool for improved understanding of heart valve hemodynamics that quantify detailed flow velocities and turbulent stresses to complement Doppler measurements. This combined information can assist in choosing optimal prosthesis for individual patients, aiding in the development of improved valve designs, and illuminating subtle changes to help guide more timely early intervention of valve dysfunction. In this computational study, flow characteristics around a bileaflet mechanical heart valve were investigated. The study focused on the hemodynamic effects of leaflet immobility, specifically, where one leaflet does not fully open. Results showed that leaflet immobility increased the principal turbulent stresses (up to 400%), and increased forces and moments on both leaflets (up to 600% and 4000%, respectively). These unfavorable conditions elevate the risk of blood cell damage and platelet activation, which are known to cascade to more severe leaflet dysfunction. Leaflet immobility appeared to cause maximal velocity within the lateral orifices. This points to the possible importance of measuring maximal velocity at the lateral orifices by Doppler ultrasound (in addition to the central orifice, which is current practice) to determine accurate pressure gradients as markers of valve dysfunction.

## 1. Introduction

Cardiovascular disease is the leading cause of death in the world [1]. There are more than 300,000 heart valves implanted annually worldwide [2,3], with approximately half of them being mechanical valves [4]. The bileaflet mechanical heart valves (BMHVs) is currently the most common valve given their durability and desirable hemodynamics [5]. However compared to bioprostheses, they are associated with more post-surgical complications such as thrombus and pannus formation, hemolysis, and platelet activation [6,7]. Improved understanding of mechanical valve hemodynamics may be vital for diagnostic, treatment and design improvements.

Several studies [6,8,9] investigated the etiology of insidious prosthetic valve dysfunction, showing that failure of mechanical heart valves is usually related to thrombus formation and tissue overgrowth. The time interval between the valve replacement and obstruction is very broad (from 6 weeks to 13 years) and some patients with significant prosthetic valve obstruction may be completely asymptomatic long before a diagnosis is made [6]. It is often difficult to distinguish between a normally functioning BMHV and a dysfunctional BMHV with mild severity, which unfortunately can still cause life-threatening sequela in the short-term [8]. Montorsi et al. [10] found that 35% of patients had normal Doppler study despite fluoroscopy showing significant restriction in one of the leaflets. They also concluded that the distinction between blocked and hypomobile leaflet is vital. Accordingly, a great deal of research has been performed on aortic and mitral heart valves in normal function and in various states of malfunction [6,9,11] ranging from slightly restricted opening to total occlusion of one leaflet including 25%, 50%, 75%, 100% dysfunctions [2,7,12,13]. Pibarot et al. [8] reported that the increase of Doppler gradients caused by dysfunction of the valve may underestimate the true hemodynamic changes [12]. Clinicians often opt for early surgical intervention since the surgical complication rate is relatively low while valve dysfunction can lead to rapid cardiovascular collapse even with minimal or absent symptoms [6]. But controversy remains whether patients with an obstructed valve should be managed by valve replacement [14], mechanical declotting [15] or nonsurgical thrombolysis [16].

Computational fluid dynamics (CFD), along with fluoroscopic or Doppler measurements, have the potential to provide clinically important insights by providing unprecedented hemodynamic detail for prosthetic heart valves [17]. For example, analysis of blood flow characteristics such as velocity, vortex formation, and turbulent stresses, especially around the valve hinge regions [18,19,20,21] can help identify conditions that may increase the risk of blood cell damage [22,23,24]. Critical turbulent shear stress thresholds of 400 N·m^−2^ [25] and 800 N·m^−2^ [26] for blood cell damage were reported. Studies also showed that high turbulent shear stress levels at the valve hinges and downstream of the valve can lead to thrombus formation and leaflets motion restriction [27,28]. This, in turn, may lead to a life-threatening dysfunction of one or both leaflets of BMHVs [12]. Fortunately, prompt recognition of valve dysfunction allows early treatment [8], and many potential complications can be prevented or minimized with careful medical management and periodic monitoring of valve function; e.g., blocked leaflets could be fully recovered when valve thrombosis is detected early [10]. CFD may also provide valuable information to speed up the design of implantable devices during the prototype development [29] and reduce the costs and risks associated with new heart valve designs [30]. Hence, analysis of flow dynamics and the resulting turbulence [31,32,33,34] and sounds [35,36,37,38,39,40] has been an active area of research.

The current computational study provides new quantitative information on blood flow characteristics, plus forces and moments acting on the leaflets of bileaflet mechanical heart valves at different levels of leaflet dysfunctionality during peak systolic flow. Model improvements compared to previous studies include: A more realistic aortic sinuses geometry (compared to References [41,42]), addition of the valve ring to the model (compared to References [43,44]), and creation of a 3-D model instead of a 2-D model (compared to References [2,13,45]). The study quantified important hemodynamic characteristics (such as principle stresses) that are not measurable using currently available standard diagnostic tools. This approach may provide a patient-specific tool for identification of adverse conditions that are associated with increased risk of hemolysis and thrombus formation [46,47], thereby potentially providing a more complete picture of the valve status useful in clinical management of patients with dysfunctional valves. The current CFD study focused on a geometric representative of leaflet dysfunction, which provided condition-specific hemodynamic changes. Patient-specific information can be obtained by carrying out similar CFD studies for actual geometries extracted from medical imaging modalities.

## 2. Materials and Methods

**Figure 1:**
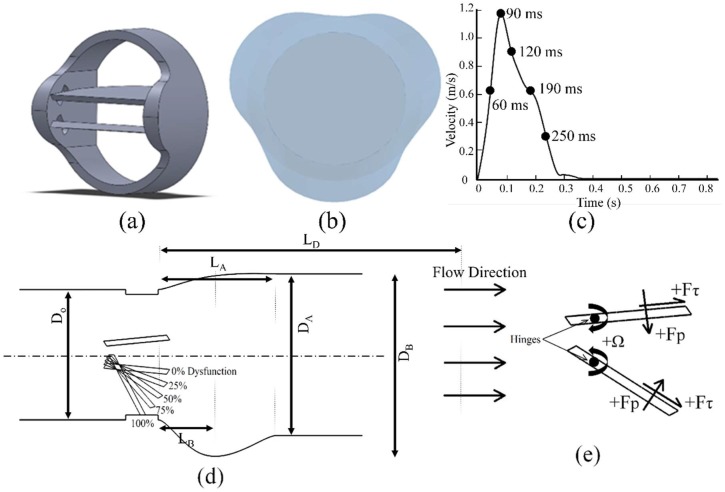
The geometry, inlet conditions and sign convention used in the current study: (**a**) Valve geometry; (**b**) Cross-section of the aortic root sinuses; (**c**) Inlet velocity profile; (**d**) Degrees of bottom leaflet dysfunction; and (**e**) Sign conventions for forces acting on the leaflets.

In this study, the computational domain was divided into four sequential regions in the flow direction: Upstream, BMHV, aortic sinuses and downstream. The heart valve geometry (Figure 1a) was chosen to be similar to previous studies [48,49]. A realistic geometry of the aortic sinuses was created since this is important for appropriate flow field analysis [50,51]. Another enhancement implemented in the current study (compared to some previous two-dimensional CFD studies) was to include the valve ring into the model. Figure 1b shows the asymmetric aortic sinuses geometry with inlet aortic root diameter of D_O_ = 23 mm, which was extracted from angiograms [52]. In this paper, the aortic root was modeled based on following parameters [52]: D_O_ = 22.3 mm is the diameter of aortic annulus, D_A_ = 27.7 mm is aortic diameter, D_B_ = 34.6 mm is the maximum projected sinus diameter, L_A_ = 22.3 mm is the length of the sinuses, and L_B_ = 7.6 mm is the distance between D_O_ and D_B_ (from the entrance of the aortic sinuses to the middle of the sinuses with the maximum projected sinus diameter), as described in Figure 1d. These parameters can be computed based on the aortic annulus diameter (D_O_), which is the same as the size of the implanted mechanical heart valve. L_D_ = 100 mm is the length of the region downstream of the heart valve. Here, the BMHV is in the fully open position and divides the flow into three orifices: Two of them (top and bottom orifices) are roughly semicircular and the third (middle orifice) is approximately rectangular.

The CFD analysis was performed for a pulsatile flow through a three-dimensional BMHV during one cardiac cycle. The analyses were focused on the period from 60 to 250 ms, where the leaflets are expected to be fully open [30]. Some results concentrated on the peak systole (90 ms), as the highest flow fluctuations, pressure gradient, and turbulent stresses associated with high risk of blood damage and platelet activation could occur at this time. To reproduce a physiological flow waveform through the aortic heart valve, the following properties were obtained from recent and previous experimental and numerical studies [2,41,53]. The inlet velocity corresponded to cardiac output of 5 L·min^−1^ and heart rate of 70 bpm with a systolic phase duration of 0.3 s (Figure 1c). The peak inflow velocity was about 1.2 ms^−1^. The density and dynamic viscosity of blood were set to ρ = 1080 kg·m^−3^ and μ = 0.0035 Pa·s, respectively. This corresponds to an inlet peak Reynolds number (Repeak=ρUpeakdinletμ) of 8516 and a Womersley number (Wo=d2ωρμ)=26.5; where, ω=2πT=17.21 rad·s−1, is the frequency of pulsatile flow and T =0.866 s is the period.

In the current study, a normal functioning (i.e., 0% dysfunction) and a BMHV with different levels of dysfunction were simulated using a commercial CFD software package (STAR-CCM+, CD-Adapco, Siemens PLM, Plano, TX, USA). Figure 1d shows the side cross section of the BMHV with a top functional leaflet and a bottom dysfunctional leaflet at 0, 25, 50, 75 and 100% levels of dysfunctionality (corresponding to a gradually decreasing effective orifice area (EOA)). In addition, Figure 1e shows the leaflet hinges as well as the direction of net pressure, shear forces (F_p_ and F_τ_, respectively), and moments (Ω) acting on the leaflets. The positive direction of the F_p_ and moments acting on both leaflets are in the direction tending to open the leaflets.

**Figure 2:**
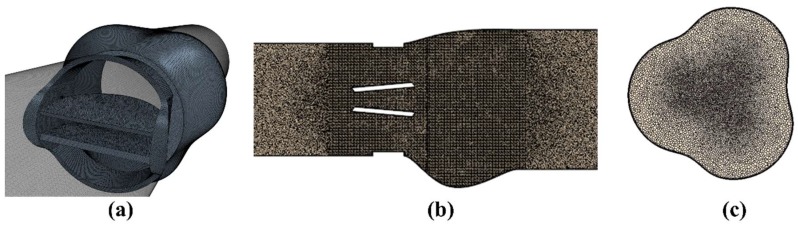
High quality mesh generated (**a**) close to the wall and leaflet surfaces; (**b**) in the flow domain; and (**c**) cross-sectional view of the mesh in the aortic root sinuses region downstream of the heart valve.

The Wilcox’s standard-Reynolds k-Omega turbulence model [43,54], which is known to perform well for internal flows, was used to simulate the flow during a complete cardiac cycle. The current and other studies [2,55] focus on the period from 60 to 250 ms, where the leaflets are expected to be fully open [30]. Hence, the dynamics of the leaflet opening and closure were not simulated as done in previous studies [2,30,43], which lowers computational cost. Therefore, the valve leaflets were assumed to remain fully open throughout the forward flow phase, which was considered reasonable because the opening and closing motions occur quickly compared to the total opening time. The unsteady simulation was performed with a time step of 0.5 ms and 25 iterations per time step. Numerical solution typically converged to residuals about <10^−4^. Moreover, high quality polyhedral mesh was generated in the flow domain, especially in the heart valve and aortic sinuses regions (Figure 2). y^+^ was maintained at less than 1 close to all walls including leaflet surfaces ($y^{+}=0.46$ at the peak flow).

### 2.1. Numerical Uncertainty

**Figure 3:**
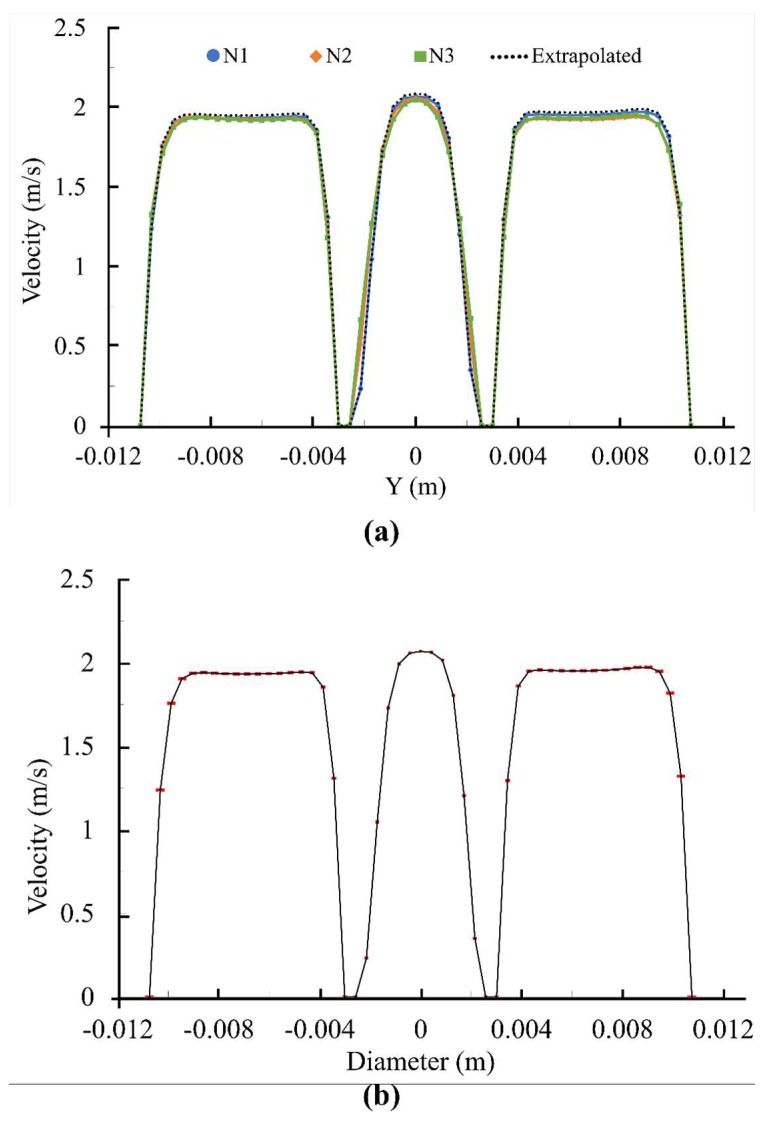
(**a**) Velocity profile at the entrance of the aortic sinuses for different grid solution; (**b**) Fine-grid solution with discretization error bars.

Steady flow simulation was conducted to establish grid density prior to unsteady simulation. The uncertainty and error in the study was calculated following ASME recommendations [56]. Figure 3 shows velocity profile at the entrance of the aortic sinuses along with the corresponding error bars while Table 1 shows the discretization error of the maximum velocity value in the entire field. The fine-grid convergence index (GCI_fine_) in Table 1 was 0.139% (excluding modeling errors [56]). In addition, the maximum discretization uncertainty was approximately 7% in the area close to the leaflets. These numerical uncertainties are comparable to previous studies [2].

### 2.2. Validation

**Figure 4:**
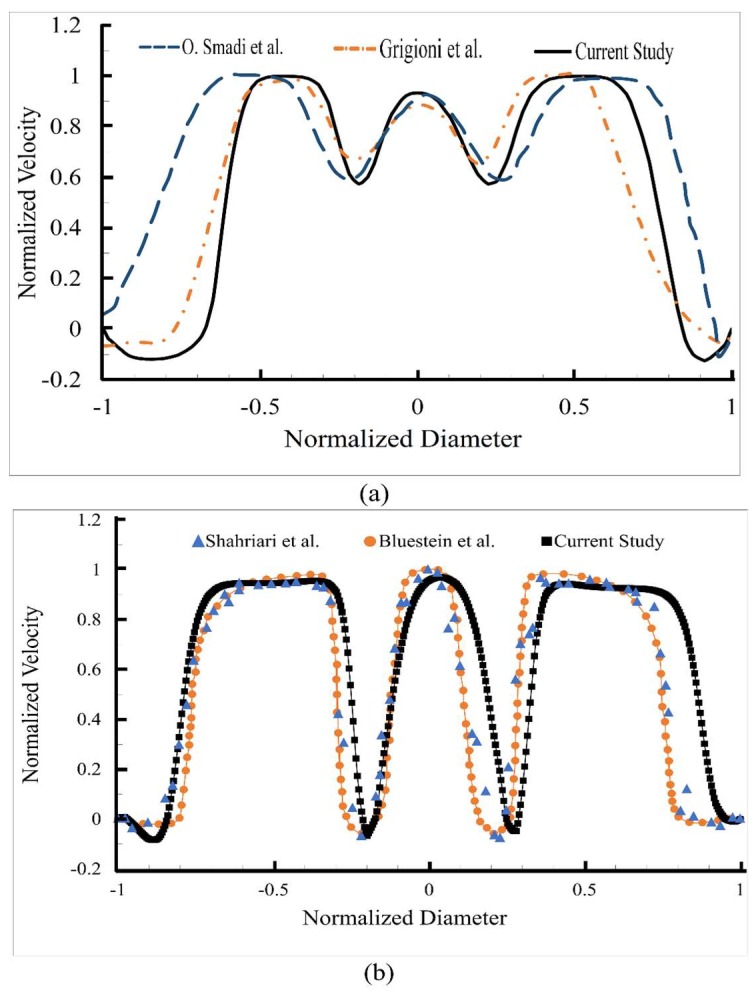
(**a**) Normalized velocity profiles at 7 mm downstream of the valve (at the peak systole) in the current study compared to previous experimental [53] and computational [13] studies. More agreement can be seen between the current and the experimental study; (**b**) Normalized velocity profiles at the trailing edge of the leaflets (105 ms after the peak systole) in the current study compared to previous experimental [43] and computational [7] studies.

The normalized velocity profile along a line located 7 mm downstream of the healthy valve (at the peak systole) is shown in Figure 4a for a normal functioning valve. The velocity profiles obtained in previous studies that considered similar geometries and flow conditions [13,53] are also shown in the same figure. Here, normalized velocities are plotted to facilitate comparison with studies that reported normalized profiles [13]. The maximum velocities were compared for steady cardiac outputs of 5 and 7 L·min^−1^. These velocities were 0.96 ms^−1^ and 1.35 ms^−1^ in the current study, respectively, which were comparable to maximum velocities of 1.0 ms^−1^ and 1.36 ms^−1^ reported in the previous study [13]. To quantify the difference between our computational results and the previous experimental results [53], the root-mean-square (RMS) of the velocity differences between the two studies were calculated. The RMS of the velocity difference was 6.58% of the maximum velocity, suggesting agreement between the results of the current study and measured values. The normalized velocity profile was also compared with two other experimental and computational studies at the trailing edge of the leaflet and 105 ms after the peak systole [7,43] (Figure 4b). The RMS of the velocity difference was <6% of the maximum velocity, suggesting agreement with these studies.

## 3. Results and Discussions

**Figure 5:**
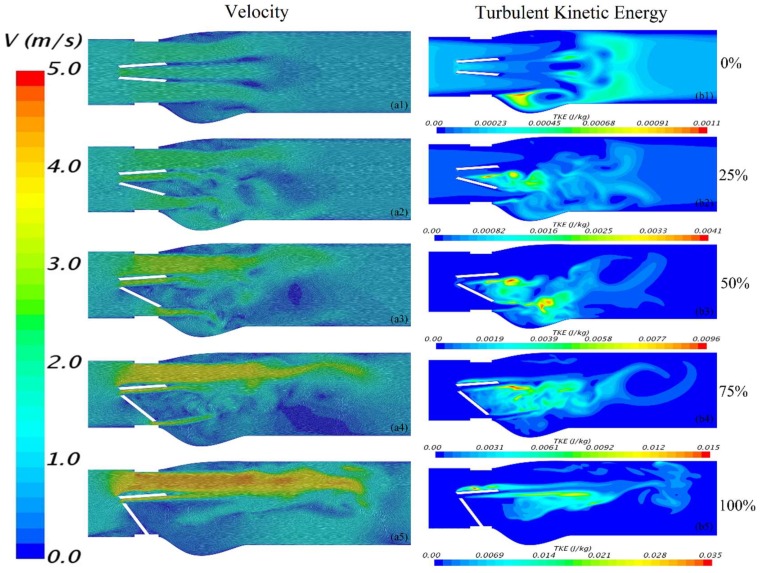
Velocity (a1–a5) and turbulent kinetic energy (b1–b5) at 90 ms for different degrees of lower leaflet dysfunction. There was a general trend of increased maximum velocity and TKE with increased dysfunction. (Note that the scale for TKE was increased with dysfunction).

Figure 5a shows a cross-sectional view of the velocity at t = 90 ms, where the color represents the magnitude and the short lines indicate direction. For 0% dysfunction (Figure 5(a1)), the flow was more uniform; especially compared to cases with dysfunctional leaflets (Figure 5(a2–a5)). Figure 5(a1) also shows a relatively small increase in velocity in the orifices and wake regions downstream of the leaflets as would be expected. As the bottom leaflet dysfunction took place, the velocity magnitude in the orifices increased. This is likely because of the narrowing of bottom orifice with dysfunction, which led to flow area reduction. Flow separation in the middle orifice was observed around the leading edge of the bottom leaflet for dysfunctionalities of 25–100% (Figure 5(a2–a5)). Separation also occurred close to the trailing edge of the top leaflet for 75% and 100% (Figure 5(a4,a5)). In addition, Figure 5a shows a trend of increasing separation bubble size with dysfunctionality. Although not clearly shown in the figure, vortex shedding was also observed. While Figure 5 shows information for t = 90 ms, flow structures were also examined for all times between 60 to 250 ms and were found similar to those shown in Figure 5.

Figure 5b shows the turbulent kinetic energy (TKE), which is an indicative of velocity fluctuations. TKE tended to increase with dysfunction and a region of higher TKEs (up to 150% compared to the healthy valve) around the top leaflet started to develop when dysfunction reached ≥75%.

**Figure 6:**
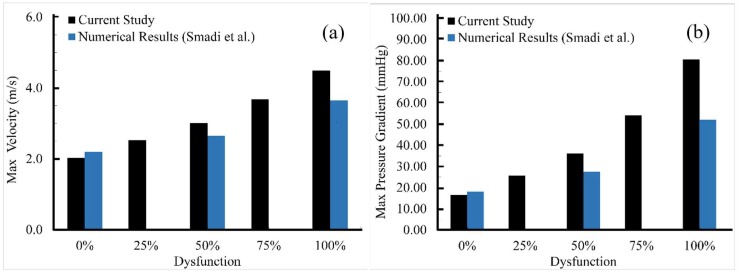
Comparison of the current study results with available data from a previous computational study [2]: (**a**) Maximum velocity at the entrance of the aortic sinuses, and (**b**) maximum pressure gradients across the valve computed from simplified Bernoulli equation. Both quantities continuously increased with dysfunction. While the trends were similar, differences may be due to the geometrical variations and the fact that the current study performed 3D compared to 2D simulation.

Figure 6a shows the maximum velocities at the entrance of the aortic sinuses, which were comparable to a previous computational study in which the results for only three dysfunctional cases (0%, 50%, and 100%) were reported [2]. In the current study, the maximum velocity increased from 2.05 ms^−1^ to 4.49 ms^−1^ as dysfunction increased from 0% to 100%. The highest velocity elevation was likely associated with the jet that originates from the orifice between the healthy leaflet and the valve ring and not from the center orifice between the two leaflets. However, when velocity gradients are measured using Doppler, it is more common that that velocity at the center orifice is measured. The smaller peak velocities that may be detected at the center orifice can lead to false estimation of velocity and pressure gradients, which can translate into errors in in assessing the severity level of leaflet dysfunction [8].

It is also to be noted that the maximum transvalvular pressure gradient (TPG_max_) can be computed from the maximal instantaneous velocity using the simplified Bernoulli equation (TPG_max_ = 4v^2^_max_) [12]. Figure 6b shows the maximum pressure gradient compared to the previous study [2] for different levels of dysfunction. Here, the TPG_max_ increased from 16.48 to 80.64 mmHg. The higher velocities and pressure gradients in the current study can be because of the smaller valve diameter and the addition of valve ring (which likely caused more flow obstruction).

**Figure 7:**
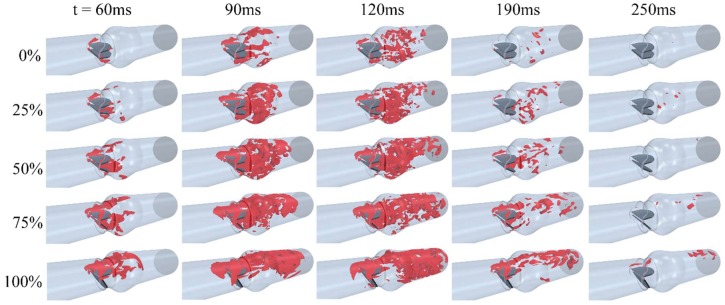
Helicity isosurfaces (isovalue = 414 m.s^−2^) at different times and dysfunctions. A general increase in helicity was observed with dysfunction.

Figure 7 shows helicity isosurfaces at different times and dysfunction levels. Since helicity is proportional to the flow velocity and the vorticity, it indicates the potential for development of helical flow. The data in this figure showed that helicity increased with dysfunction and peaked around peak systolic velocity time. Figure 7 also suggested that intense vortical structures start to appear in the valve and sinus regions during the acceleration phase (e.g., 60 ms) before spreading downstream at later times. For leaflet dysfunction of ≥75%, lower helicity (compared to dysfunctionality of <75%) was observed in the dysfunctional leaflet side, which can be because the region downstream of that leaflet may contain lowered velocity and vorticity.

Several studies reported that the hemolysis (the breakage of a red blood cell membrane), can occur for turbulent shear stresses in the range from 400 to 5000 N·m^−2^ with exposure time as small as 10 ms [15,51]. In addition, these high turbulent shear stresses can lead to platelets activation, which increase the risk of platelet aggregation and blood clots formation [10,15]. Clots may detach and the resulting free-floating clot can block arteries leading to serious consequences such as embolism and stroke [26].

While stresses acting on the fluid occur in different directions, principal stresses are the highest. Three-dimensional principal stress analysis requires the computation of the full Reynolds stress tensor (*T*):

(1) $$T=\left[\begin{array}{ccc} \sigma_{xx} & \tau_{xy} & \tau_{xz}\\ \tau_{yx} & \sigma_{yy} & \tau_{yz}\\ \tau_{zx} & \tau_{zy} & \sigma_{zz} \end{array}\right]=\rho \left[\begin{array}{ccc} \bar{uu} & \bar{uv} & \bar{uw}\\ \bar{vu} & \bar{vv} & \bar{vw}\\ \bar{wu} & \bar{wv} & \bar{ww} \end{array}\right]\;$$

where, *u*, *v*, and *w* are the velocity fluctuation components and, σ and τ represent normal and shear stresses, respectively. Popov [57] provides a detailed discussion of the calculation of three-dimensional maximum or principal stresses which involves the solution of the roots of the following third order equation:

(2) $$\sigma^{3}-I_{1}\sigma^{2}+I_{2}\sigma -I_{3}=0$$

where,

(3) $$I_{1}=\sigma_{xx}+\sigma_{yy}+\sigma_{zz}$$

(4) $$I_{2}=\sigma_{xx}\sigma_{yy}+\sigma_{yy}\sigma_{zz}+\sigma_{xx}\sigma_{zz}-\tau_{xy}{}^{2}-\tau_{yz}{}^{2}-\tau_{xz}{}^{2}$$

(5) $$I_{3}=\sigma_{xx}\sigma_{yy}\sigma_{zz}+2\tau_{xy}\tau_{yz}\tau_{xz}-\sigma_{xx}\tau_{yz}{}^{2}-\sigma_{yy}\tau_{xz}{}^{2}-\sigma_{zz}\tau_{xy}{}^{2}\;$$

The three roots $\sigma_{1}<\sigma_{2}<\sigma_{3}$ of the above equation are the three principal normal stresses. The coefficients $I_{1},\;I_{2}$ and $I_{3}$ are functions of the measured Reynolds stress tensor and are the three stress invariants of the Reynolds stress tensor. In addition, the maximum or principal shear stresses $(\tau_{ij}{}_{P}$) are linearly related to the normal stresses by the following equations:

(6) $$\tau_{ij}{}_{P}=\frac{\sigma_{i}-\sigma_{j}}{2};\;\tau_{max}=\frac{\sigma_{3}-\sigma_{1}}{2}$$

**Figure 8:**
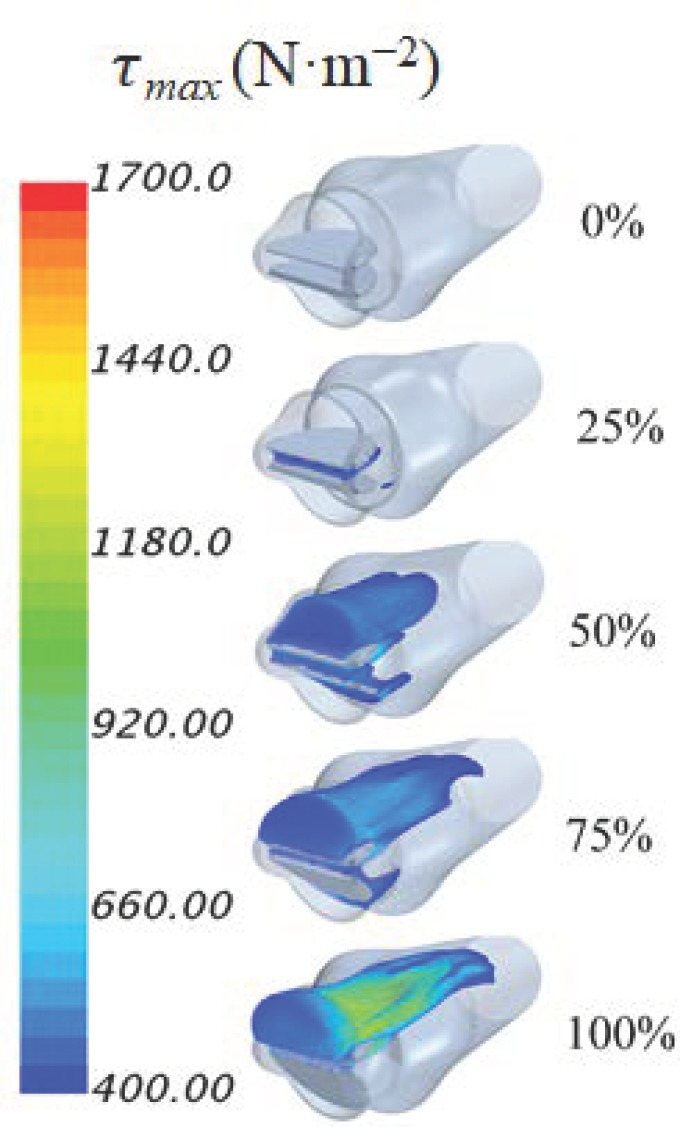
Principal shear stresses for different levels of dysfunction at the peak systole. Elevated levels of principal stresses were observed with dysfunction, which increase blood damage risks. Published cutoff stress value for damage is above 400 N·m^−2^ [25].

Figure 8 displays turbulent shear ($\tau_{max}$) principal stresses for different levels of dysfunction at the peak systole. Since an increased risk of blood damage may occur for stresses exceeding 400 N·m^−2^, only stresses in this range are shown. These results suggested that as the leaflet dysfunctionality increased, the principal turbulent shear stresses increased. More specifically for 0 %, 25%, 50%, 75%, and 100% dysfunction levels, the maximum principal shear stresses at peak systole were 420, 510, 760, 1155, and 1695 N·m^−2^. In addition, the regions of elevated stresses grew with dysfunction and were concentrated around and downstream of the functional (top) leaflet where high jet velocity and stronger helical structures existed (Figure 5 and Figure 7). These regions are of the particular interest since elevated turbulent stress levels are known to be associated with blood damage and thrombus formation. In addition, careful examination of Figure 8, indicates that the increase in the region with high principal stresses accelerates later (>50%) for the current model. While this ~50% threshold may vary with geometry, CFD will allow patient-specific analysis, which may further increase its utility. Future investigations of other realistic geometries may be performed to quantify this effect.

The highest principal turbulent stresses, however, occurred slightly after (100–120 ms) peak systole during the deceleration phase. Table 2 shows the highest principal turbulent stress values and their occurrence time. It can be seen that these values were somewhat higher (~4–14%) than those at peak systole. Comparing to previous experimental studies, lethal and sublethal damages of red cells can occur with turbulent shear stresses as low as 150 and 50 N·m^−2^, respectively [46,58]. These levels can be significantly lower (1–10 N·m^−2^) in the presence of foreign surfaces such as valve prostheses [59,60]. In addition, platelet activation can occur when turbulent shear stresses are in the range of 10–50 N·m^−2^ [46,47]. Studies also showed that high turbulent shear stresses at the valve hinges and downstream of the valve, for normal cases (valve leaflet with 0% dysfunction), can lead to thrombus formation and the leaflets’ motion restriction [27,28]. This, in turn, may lead to a life-threatening dysfunction of one or both leaflets of BMHVs [12].

**Figure 9:**
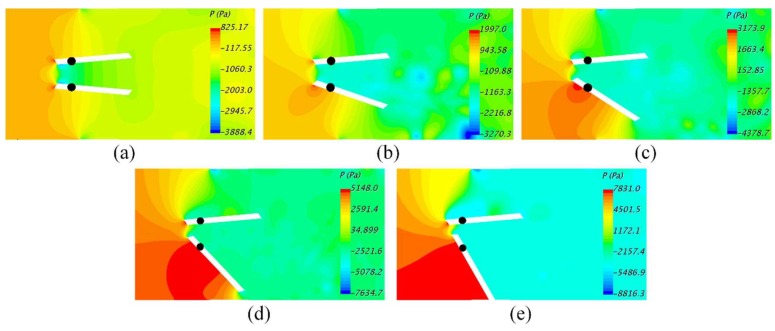
(**a**) 0%; (**b**) 25%; (**c**) 50%; (**d**) 75%; and (**e**) 100%. For dysfunction ≥ 75%, a region of high pressure developed at the bottom surface of the functional leaflet upstream of the hinge, which would generate moments that tend to keep that leaflet open.

Figure 9 shows the pressure distribution in the vicinity of the leaflets. The maximum pressure at the blocked leaflet increased with dysfunction. For dysfunctions higher than 50%, a region of high pressure developed at the bottom surface of the functional leaflet upstream the hinge, which would generate higher moments in the direction of leaflet opening.

**Figure 10:**
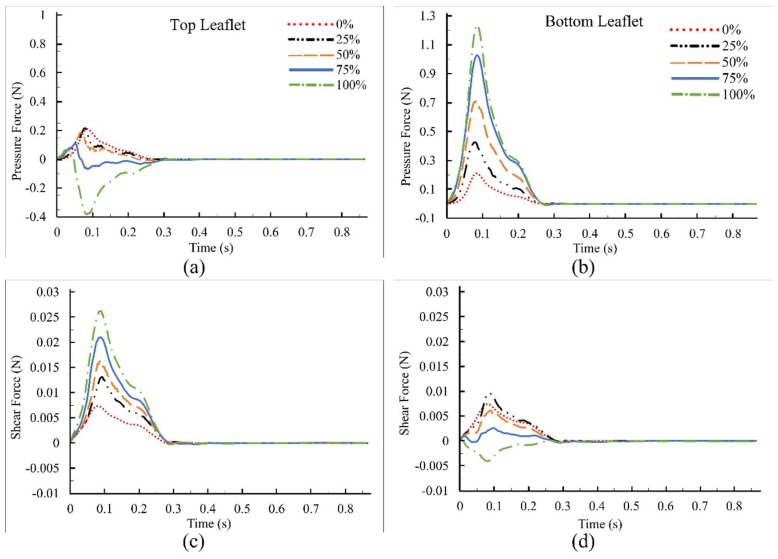
Net pressure and shear forces on leaflets: (**a**) F_p_ on top leaflet; (**b**) F_p_ on bottom leaflet; (**c**) F_τ_ on top leaflet; and (**d**) F_τ_ on bottom leaflet. The sign of some forces started to reverse at high levels of dysfunction. The legends are consistent for all four figures.

**Figure 11:**
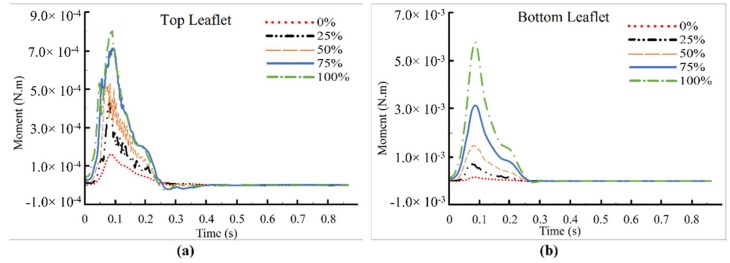
Net moments on: (**a**) Top leaflet, and (**b**) Bottom leaflet. The moments tended to be in the directions of leaflet opening. All moments increased with dysfunction. In most cases of dysfunction, the moments on the dysfunctional leaflet were higher (note the different scale for the dysfunctional leaflet).

It is important to document elevated forces and moments, as they would lead to higher reaction forces at the hinges (where thrombus tends to form), which may create more adverse conditions. Collection and analysis of this information can also aid in the development of improved valve designs. The net pressure and shear forces on the top and bottom leaflets for the full cardiac cycle are displayed in Figure 10. Results showed that increased dysfunctionality of one leaflet led to higher net forces on the functional and dysfunctional leaflets up to 200%, and 600%, respectively. Note that although the net pressure forces (F_p_) on the top leaflet were negative (upward) for 75% and 100% dysfunctions, forces were acting upstream of the hinges (Figure 9d–e), which would result in positive moments (Figure 11a). Figure 10b shows the F_p_ on the bottom leaflet, which was positive for all cases. Net shear forces (F_τ_) on the top and bottom leaflets (Figure 10c and Figure 10d, respectively) were positive during the period under consideration for all levels of dysfunction except for the dysfunctional leaflet with 100% dysfunction. The change in the sign may be attributed to the large revered flow regions (Figure 5a and Figure 9) that formed downstream of the leaflet, as resulted in positive moments on bottom leaflet (Figure 11b).

Future CFD studies can explore new heart valve designs and structural materials and determine how blood-material interactions and hemodynamics can be affected by design changes [61] with the aim of reducing thrombo-embolic complications associated with these valves, which can lead to improved valve designs. For example, analysis of blood flow characteristics through a BMHV especially around the valve hinge regions can help identify conditions that may increase the risk of blood cell damage [22,23]. An investigation of the effect of the leaflet opening angles on the blood flow also suggested that the opening angle can highly affect the flow downstream of BMHV and that opening angles >80 degrees would be more effective in reducing flow resistance and vortical structures [62].

## 4. Conclusions

In this study, adverse hemodynamic conditions at peak systole due to incomplete leaflet opening of a bileaflet mechanical heart valve were investigated. A realistic 3-D geometry of the aortic sinuses and a complete model of a bileaflet mechanical heart valve including the valve ring were constructed. The results suggest that maximum blood velocities increased when the effective orifice area was reduced due to the increase of leaflet dysfunction, as expected. Leaflet immobility also appears to cause maximal velocity within the lateral orifices. This points to the possible importance of measuring maximal velocity at the lateral orifices by Doppler ultrasound (in addition to the central orifice which is current practice) to determine accurate pressure gradients as markers of valve dysfunction. Dysfunctionality also increased the transvalvular pressure gradient by up to 300%, which would increase the effort to produce the same cardiac output.

Results also suggested that the higher levels of dysfunction were accompanied with flow separation at the leaflet surfaces and growing eddies especially downstream of the valve in the aortic sinuses. Principal turbulent stresses for immobile leaflet increased up to 1695 N·m^−2^, which exceeds the threshold values for elevated risk of hemolysis and platelet activation and lead to potential development of thrombosis, especially around the normal leaflet. The region with high principal stresses (i.e., above threshold = 400 N·m^−2^) initially grew slowly (i.e., between 0 and 25% dysfunction), and then covered a significantly large region at higher dysfunctions (i.e., >50% of leaflet dysfunction), Figure 8, suggesting a possible need for closer monitoring of the patients with >50% of leaflet dysfunction. Dysfunctionality of one leaflet led to higher net forces on the leaflets (by up to 200%, and 600% for healthy and the dysfunctional leaflets, respectively). The resulting moments acting on the leaflets also increased with dysfunctionality (up to 550%, and 4000% for healthy and dysfunctional leaflets, respectively). These higher forces and moments can increase the reaction forces and stresses in the hinge region where vulnerability to thrombus and pannus formations tend to be high and can lead to more leaflet motion restriction.

## Figures and Tables

**Table 1:** Calculation of discretization error.

*φ* = Maximum Velocity in the Entire Field (m/s)			
N_1_; N_2_; N_3_ 6,529,062; 2,598,513; 1,390,150			
r_21_ (Refinement factor of N_2_/N_1_)	1.35	**e** ^21^_a_	0.11%
r_32_ (Refinement factor of N_3_/N_2_)	1.32	**e** ^21^_ext_	0.11%
*φ*_1_	2.523	**GCI** ^21^_fine_	0.14%
*φ*_2_	2.521	*φ* ^32^_ext_	2.515
*φ*_3_	2.526	**e** ^32^_a_	0.21%
P	2.289	***e*** ^32^_ext_	0.24%
*φ* ^21^_ext_	2.526	**GCI** ^32^_course_	0.29%

**Table 2:** Maximum Principal Shear Stresses.

Dysfunction	Max. Principal Shear Stress (N·m^−2^)	Time (s)
0%	440	0.102
25%	534	0.103
50%	832	0.112
75%	1276	0.112
100%	1972	0.119
